# Validation studies on migraine diagnostic tools for use in nonclinical settings: a systematic review

**DOI:** 10.1055/s-0042-1756490

**Published:** 2022-10-27

**Authors:** Du Wei, Li Ping Wong, Tharani Loganathan, Rong-Rui Tang, Yue Chang, Han-Ni Zhou, Mohammed K. A. Kaabar

**Affiliations:** 1 Universiti Malaya, Faculty of Medicine, Department of Social and Preventive Medicine, Kuala Lumpur, Malaysia.; 2 Guizhou Medical University, School of Medicine and Health Management, Guiyang, China.; 3 University-Town Hospital of Chongqing Medical University, Department of Neurosurgery, Chongqing, China.; 4 Universiti Malaya, Faculty of Science, Institute of Mathematical Sciences, Kuala Lumpur, Malaysia.; 5 Washington State University, Pullman, Department of Mathematics and Statistics, Washington, United States.

**Keywords:** Migraine Disorders, Diagnosis, Sensitivity and Specificity, Systematic Review, Transtornos de Enxaqueca, Diagnóstico, Sensibilidade e Especificidade, Revisão Sistemática

## Abstract

**Background** Migraine underdiagnosis and undertreatment are so widespread, that hence is essential to diagnose migraine sufferers in nonclinical settings. A systematic review of validation studies on migraine diagnostic tools applicable to nonclinical settings can help researchers and practitioners in tool selection decisions.

**Objective** To systematically review and critically assess published validation studies on migraine diagnostic tools for use in nonclinical settings, as well as to describe their diagnostic performance.

**Methods** A multidisciplinary workgroup followed transparent and systematic procedures to collaborate on this work. PubMed, Medline, and Web of Science were searched for studies up to January 17, 2022. The QUADAS-2 was employed to assess methodological quality, and the quality thresholds adopted by the Global Burden Disease study were used to tail signaling questions.

**Results** From 7,214 articles identified, a total of 27 studies examining 19 tools were eligible for inclusion. There has been no high-quality evidence to support any tool for use of migraine diagnosis in nonclinical settings. The diagnostic accuracy of the ID-migraine, structured headache and HARDSHIP questionnaires have been supported by moderate-quality evidence, with sensitivity and specificity above 70%. Of them, the HARDSHIP questionnaire has been the most extensively validated. The remaining 16 tools have provided poor-quality evidence for migraine diagnosis in nonclinical populations.

**Conclusions** Up till now, the HARDSHIP questionnaire is the optimal choice for diagnosing migraine in nonclinical settings, with satisfactory diagnostic accuracy supported by moderate methodological quality. This work reveals the crucial next step, which is further high-quality validation studies in diverse nonclinical population groups.

## Resumo

**Antecedentes** O sub-diagnóstico e o subtratamento da enxaqueca são tão difundidos que, portanto, é essencial para diagnosticar os portadores de enxaqueca em ambientes não-clínicos. Uma revisão sistemática dos estudos de validação das ferramentas de diagnóstico da enxaqueca aplicáveis a ambientes não-clínicos pode ajudar os pesquisadores e profissionais nas decisões de seleção de ferramentas.

**Objetivo** Revisar sistematicamente e avaliar criticamente estudos de validação publicados sobre ferramentas de diagnóstico da enxaqueca para uso em ambientes não-clínicos, bem como descrever seu desempenho diagnóstico.

**Métodos** Um grupo de trabalho multidisciplinar seguiu procedimentos transparentes e sistemáticos para colaborar neste trabalho. PubMed, Medline e Web of Science foram pesquisados por estudos até 17 de janeiro de 2022. O QUADAS-2 foi empregado para avaliar a qualidade metodológica, e os limites de qualidade adotados pelo estudo da Global Burden Disease foram usados para responder a questões de sinalização.

**Resultados** De 7.214 artigos identificados, um total de 27 estudos examinando 19 ferramentas foram elegíveis para inclusão. Não houve evidência de alta qualidade para apoiar qualquer ferramenta para o uso de diagnóstico de enxaqueca em ambientes não clínicos. A precisão diagnóstica do ID-Migraine, questionário de dor de cabeça estruturada e questionário HARDSHIP foram apoiados por evidências de qualidade moderada, com sensibilidade e especificidade acima de 70%. Deles, o questionário HARDSHIP foi o mais amplamente validado. As 16 ferramentas restantes forneceram provas de má qualidade para o diagnóstico de enxaqueca em populações não-clínicas.

**Conclusões** Até agora, o questionário HARDSHIP é a escolha ideal para o diagnóstico da enxaqueca em ambientes não-clínicos, com precisão diagnóstica satisfatória apoiada por uma qualidade metodológica moderada. Este trabalho revela o próximo passo crucial, que é a realização de mais estudos de validação de alta qualidade em diversos grupos populacionais não-clínicos.

## INTRODUCTION

Migraine ranks as the second leading cause of disability worldwide according to the 2017 Global Burden of Disease (GBD) study. 1 Even though migraine does not cause death, 2 this condition leads to 45.1 million (95% uncertainty interval [UI]: 29 to 62.8) disability-adjusted life years (DALYs) each year, and is responsible for 599 (95% UI: 386 to 833) per 100,000 population of age-standardized DALY rate. 3 That is equivalent to 45.1 million years of healthy life lost each year. It has been estimated that approximately 2% of the gross domestic product globally is lost annually due to migraines. 4 However, despite the debilitating effects of migraines, more than half of migraine patients have never consulted a medical practitioner, 5 and more than two-thirds have not received any treatments. 6

Therefore, considering the low disease awareness, it is essential to allow more patients to be diagnosed in nonclinical settings. Several systematic reviews of migraine identification tools have been published, but their inclusion criteria are tools that support clinical decisions for primary care practitioners. 7 8 Even though advanced digital diagnostic tools such as wearable headsets and machine learning programs have appeared recently, 9 the diagnosis of migraines remains largely reliant on physician interpretation. The performance of currently available migraine diagnostic tools that are usable in nonclinical setting is unclear. We attempted to bridge this gap by conducting a systematic review and providing tool selection advice for researchers and practitioners.

Although evidence-based International Classification of Headache Disorders (ICHD) criteria are available, they are intended for professional use only. 10 This is because the technical concepts in the criteria, such as photophobia and phonophobia, are not easily understood by lay respondents. If a study is trying to apply a diagnostic tool for migraine in nonclinical settings, the tool must be validated to demonstrate that it is methodologically reasonable in comparison to the “gold standard” 11 . The “gold standard” for migraine diagnosis has been widely accepted as a clinical diagnosis made by a neurologist, based on the latest ICHD criteria after physical examinations and reviewing the patients' medical history, 12 as there has been no objective biological/instrumental marker for the diagnosis of migraine. 13

A systematic review of validation studies can aid in understanding existing evidence on diagnostic tools for use in nonclinical settings. As a result, we performed this systematic review with the objectives of 1) assessing the methodological quality of published validation studies on migraine diagnostic tools that have been reported to be usable in nonclinical settings, and 2) describing their diagnostic accuracy, including sensitivity, specificity, positive predictive value (PPV), or negative predictive value (NPV).

Following the introduction, section 2 details the methods for this systematic review. Next, section 3 is the presentation of the results. This is followed by section 4, which discusses the findings and quality issues of existing evidence. Ultimately, the conclusions on tool selection and suggestions for future work are provided.

## METHODS

We followed the Cochrane guidelines 14 for methodology, and the Preferred Reporting Items for Systematic Reviews and Meta-Analyses (PRISMA) for reporting. The obtained data was secondary; therefore, ethical approval was not required. The protocol for this systematic review was registered on the International Prospective Register of Systematic Reviews (PROSPERO), with registration ID CRD42021296848.

### Multidisciplinary workgroup

In August 2021, a workgroup that included five academics with extensive knowledge in public health, one with knowledge in statistics, and one neurologist with practical experience, was formed for this systematic review. Additionally, as supporting members, both methodologists and medical librarians were involved.

From August 2021 to April 2022, the workgroup and supporting members met at least once a week, either face to face or virtually, to conceptualize the research framework, establish objectives and eligibility criteria, search for evidence, appraise quality, integrate and analyze evidence, and conclude.

### Eligibility criteria

The inclusion and exclusion criteria are illustrated in Table 1 . We examined validation studies that focused on tools for migraine diagnosis, classification, or screening (hereinafter referred to as diagnostic tools) in nonclinical settings, whose eligible users were adults (≥ 18 years old). The “gold standard” reference was a clinical diagnosis given by a neurologist, relying on the ICHD criteria, who was blind to the tool's diagnosis.

**Table 1:** Inclusion and exclusion criteria

	Inclusion	Exclusion
**Population**	Human adults (≥ 18 years old)	Nonhumans; Humans aged < 18 years old
**Setting**	Nonclinical settings	Clinical settings
**Tool**	Tools intended to diagnose, classify, or screen for migraine, including migraine subtypes	Tools not for migraine
**Reference**	The gold standard, diagnosis by a neurologist based on the ICHD criteria	Others
**Outcome**	Reporting at least the following diagnostic accuracy outcomes: sensitivity, specificity, PPV, or NPV	No report of diagnostic accuracy
**Language**	English	Others
**Publication**	Peer-review publications	Unpublished studies, gray literature, reviews, guidelines, letters, commentaries, conference abstracts, and editorials

**Abbreviations:** ICHD, international classification of headache disorders; NPV, negative predictive value; PPV, positive predictive value.

### Information sources and search strategies

Prior to commencing, a search had been conducted to ensure that we were not unnecessarily duplicating a review that had been done by other scholars. Studies published from the foundation of the databases until January 17, 2022, were searched in three electronic databases: PubMed, Medline, and Web of Science. To avoid missing any relevant studies, subject terms from the controlled vocabulary were combined with free-text terms. The complete search strings for the three databases are mentioned in Table S1 ( Supplementary Material , available online only). Additional articles were manually located from citations and references of the included studies.

### Study screening

The Endnote 20 (Clarivate Analytics, London, UK) software was used for screening, removing duplicates, and recording. After searches and removal of duplicates, two independent reviewers (DW and HNZ), blinded to each other, decided simultaneously whether each article was meeting the aforementioned eligibility criteria via studying titles and abstracts. After the initial screening, full-text articles were reviewed by at least two workgroup members (DW and RRT). Any discrepancies between both reviewers in terms of the inclusion and exclusion of studies were resolved through a consensus after discussing with a third reviewer (LPW).

### Methodological quality assessment

The methodological quality of the included studies was appraised using the Quality Assessment of Diagnostic Accuracy Studies (QUADAS-2), which is separated into two parts: risk of bias and applicability. The signaling questions in QUADAS-2, according to its developers, should be tailored to the subject of the review. 15 The quality thresholds for population-based studies on headaches were established by the GBD studies ( Table S2 – Supplementary Material ), 16 which were then applied to tail signaling questions in the present study ( Table S3 – Supplementary Material ).

Each QUADAS-2 domain was assessed, and each study was given a rating of “high risk/concern,” “low risk/concern,” or “unclear.” For the overall rating of risk of bias or applicability, overall “low risk of bias” or “low concern regarding applicability” was given to a study that obtained “low” in all domains, and overall “at risk of bias” or “concerns regarding applicability” was given to a study that obtained “high” or “unclear” in one or more domains. 15 Furthermore, we classified “quality” into 3 groups: “high quality” (overall “low risk of bias” in combination with overall “low concern regarding applicability”), “moderate quality” (one domain receiving “high” or “unclear” risk of bias in combination with overall “low concern regarding applicability”), and “poor quality” (all other rating combinations). Two independent reviewers (DW and YC) appraised the methodological quality of included studies. To resolve any disagreements, a third (TL) was invited.

### Data collection

For the included articles, construct data collection forms were developed and piloted. Fields extracted from each study included tool characteristics (name, aim, and language), first author, year of publication, sample characteristics (sample size and participant demographics), reference standard, time interval, and diagnostic accuracy (sensitivity, specificity, PPV, and NPV). Two reviewers (DW and MKAK) registered independently for data extraction. Any disagreements were identified and resolved by another reviewer (LPW). Insufficient accuracy statistics were calculated and supplemented by the RevMan (Cochrane, London, UK) software, version 5.4, and all outcomes were double-checked and recalculated. Pooled data were demonstrated when possible.

## RESULTS

### Literature search results

**Figure 1:**
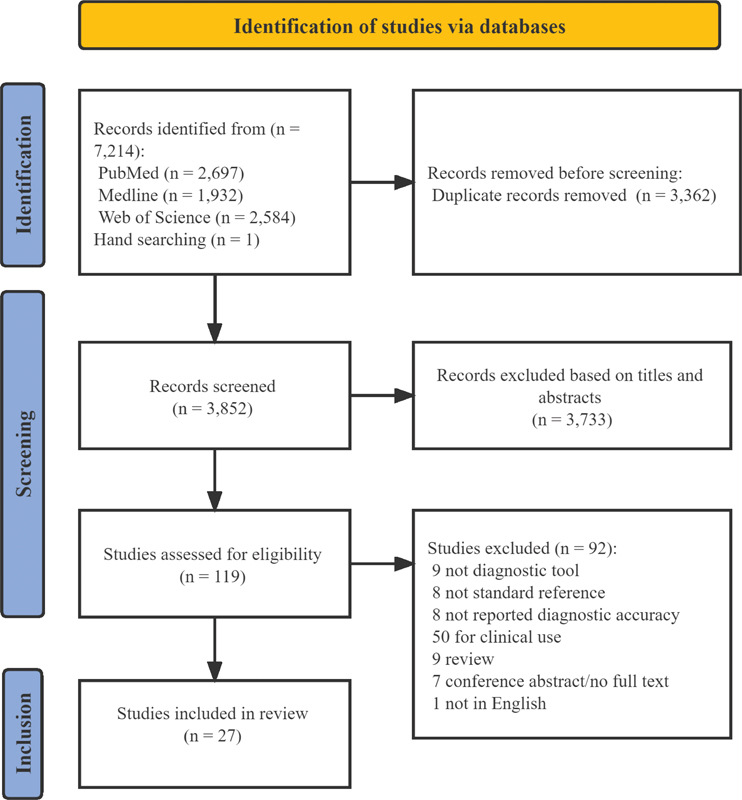
PRISMA flow diagram of the study screening process.

Figure 1 describes the PRISMA flow diagram. The search retrieved 7,213 publications, of which 3,362 duplicates were excluded. A manual search yielded 1 additional article. After determining eligibility on titles and abstracts, 119 papers remained for full-text review. Finally, 27 studies, published between 1991 and 2022, were included.

### Tool description

In total, 19 tools have been reported as being able to diagnose migraine for adults in nonclinical contexts. The studies' characteristics arranged by tool names are detailed in Table 2 . Among them, 14 tools were designed for total migraine diagnosis (ID-migraine, 17 18 19 extended version of ID-migraine, 20 MS-Q, 21 22 23 simple questionnaire, 24 Michel's standardized migraine diagnosis questionnaire, 25 diagnostic headache diary, 26 DMQ3, 27 ID-CM, 28 HUNT, 29 HUNT3, 30 HUNT4, 31 self-administered headache questionnaire, 32 HARDSHIP questionnaire, 33 34 35 36 and POEM 37 ), 9 tools for migraine with aura (extended version of ID-migraine, 20 visual aura rating scale, 38 39 DMQ3, 27 Finnish migraine-specific questionnaire, 40 LUMINA, 41 HUNT3, 30 HUNT4, 31 Italian ICHD-II-based questionnaire, 42 and POEM 37 ), and 4 tools for migraine without aura (DMQ3, 27 Finnish migraine-specific questionnaire, 40 Italian ICHD-II-based questionnaire, 42 and POEM 37 ). The structured headache questionnaire 43 and ID-CM 28 can determine chronic migraine, and the self-administered headache questionnaire 32 can recognize a combination of migraine and tension-type headaches.

**Table 2:** Characteristics of included studies

Tool	References	Aim	Translation		Sample			Reference standard	**Time interval**
			Language	Verification	Setting (Administration)	Characteristics	Selection		
ID-Migraine	Siva et al. 2008	Migraine totally	Turkish	Y	Workplace (FTF interview)	N = 227 Age: 31.9 ± 5.9 F: 65.6%	Convenience, preliminarily screen-positive	Clinical diagnosis by a neurologist based on ICHD-2	Within 1 week
	Wang et al. 2015	Migraine totally	Chinese	N	University (Not mentioned)	N = 555 Age: 22.03 ± 1.53 F: 71.7%	Volunteers and random	Clinical diagnosis or telephone interview by a neurologist based on ICHD-2	10 months
	Csépány et al. 2018	Migraine totally	Hungarian	N	Healthcare (Not mentioned)	N = 380 Age: median 36 F: 80%	Consecutive	Clinical diagnosis by a neurologist based on ICHD-3β	At the same time
Extended Version of ID- Migraine	Streel et al. 2015	Migraine totally MA	French	Original	General (Self-administered)	N = 67 Age: 20–69 F: 58.2%	Convenience	Clinical diagnosis by a neurologist based on ICHD-2	Medical history
MS-Q	Láinez et al. 2005	Migraine totally	Spanish	Original	Healthcare (Self-administered)	N = 140 Age: 39.2 ± 13 F: 73%	Volunteer	Clinical diagnosis by a neurologist based on ICHD	At the same time
	Láinez et al. 2010	Migraine totally	Spanish	No need	Healthcare (Self-administered)	N = 9,346 Age: 48.9–50.1 ± 17.2 F: 61.9%	Consecutive	Medical records by a neurologist based on ICHD-2	At the same time
	Delic et al. 2018	Migraine totally	Bosnian/Croatian/Serbian	Y	Healthcare (Self-administered)	N = 429 Age: 46–47.6 ± 12.6–12.7 F: 72.7%	Consecutive	Clinical diagnosis by a neurologist based on ICHD-3	At the same time
Simple Questionnaire	Gervil et al. 1998	Migraine totally	Danish	Original	Healthcare and family (Not mentioned)	N = 2,035	Twin census	Telephone interview by a doctor based on ICHD	1–2 years
MSMDQ	Rueda-Sánchez and Díaz-Martínez 2004	Migraine totally	Spanish	N	University (Self-administered)	N = 170	Volunteer and random	Interview by a neurologist based on ICHD	Within 2–3 weeks
SHQ	el-Sherbiny et al. 2017	EM CM	Arabic	Y	General (FTF interview)	N = 232 Age: 41.2 ± 10.9 F: 72.8%	Random	Detailed assessment by two neurologists based on ICHD-3β	After 6 months
VARS	Eriksen et al. 2005	MA	Danish	Original	Healthcare and family (FTF interview)	N = 362 patients and 108 controls	Volunteer	Telephone interview by a specially trained physician based on ICHD-2	At the same time
	Kim et al. 2022	MA	Korean	Y	Healthcare (Self-administered)	N = 240 Age: 25–50 F: 87.5%	Volunteer	Clinical diagnosis by a neurologist based on ICHD-3	At the same time
DHD	Phillip et al. 2007	Migraine totally	Danish or English	N	General (Examined by trained observer)	N = 49 Age: mean 44 (26–70) F: 75.6%	Volunteer	Clinical diagnosis or telephone interview by a neurologist based on ICHD	A maximum of 24 headache days or 2 months
DMQ3	Kirchmann et al. 2006	Migraine totally MO MA MO + MA	Danish	No need	Healthcare and family (Self-administered)	N = 147 (patients and controls) Age: median 45 (18–65) F: 74.4%	Volunteer and random	Telephone interview by a trained physician based on ICHD-2	2–3 years
FMSQ	Kallela et al. 2001	MO MA MO + MA	Finnish	Original	Healthcare and family (Self-administered)	N = 94 Age: 44.6 ± 10.9 F: 71.3%	Consecutive	Telephone interview by a neurologist based on ICHD	Not mentioned
ID-CM	Lipton et al. 2016	Migraine totally CM	English	Original	General (Self-administered)	N = 111 Age: 46.2 ± 13.4 F: 82.9%	Quota and volunteer	Telephone interview by a neurologist based on ICHD-3β	Not mentioned
LUMINA	Van Oosterhout et al. 2011	MA	English	Original	Healthcare (Self-administered)	N = 200 Age: 43.3 ± 11.5 F: 88.5%	Volunteer and random, preliminarily screen-positive	Telephone interview by a neurologist based on ICHD-2	Not mentioned
HUNT	Hagen et al. 2000	Migraine totally	Norwegian	Original	General (Self-administered)	N = 167	Volunteer and random	Clinical interview by a neurologist based on ICHD	4–8 months
HUNT3	Hagen et al. 2010	Migraine totally MA	Norwegian	No need	General (Self-administered)	N = 297 Age: mean 52.3 F: 51%	Volunteer and random	Interview + clinical diagnosis by neurologists based on ICHD-2	50 days (95% CI 48–52)
HUNT4	Hagen et al. 2019	Migraine totally MA	Norwegian	No need	General (Self-administered)	N = 232 Age: mean 58.4 F: 65.52%	Volunteer and random	Interview by neurologists based on ICHD-3	60 days (95% CI 56–62 days)
SAHQ	Fritsche et al. 2007	Migraine totally Migraine + TTH	German	No need	Healthcare (Self-administered)	Patients: N = 278 Age: mean 43.9 F: 51.1% Controls: N = 42	Case-control	Clinical diagnosis by three neurologists based on ICHD-2	At the same time
IIIBQ	Abrignani et al. 2012	MO MA (1.2.1, 1.2.2)† MA (1.2.3)†	Italian	Original	Healthcare (FTF interview)	N = 50 Age: 40.7 ± 12.8 F: 74%	Consecutive	Clinical diagnosis by one of two neurologists based on ICHD-2	On the same day
HARDSHIP	Ayzenberg et al. 2011	Migraine totally	Russian	Y	General (FTF interview)	N = 190	Random	Telephone interview by a neurologist based on ICHD-2	4–8 weeks
	Yu et al. 2011	Migraine totally	Chinese	Y	General (FTF interview)	N = 399	Random	Telephone interview by a neurologist based on ICHD-2	Not mentioned
	Rao et al. 2012	Migraine totally	Kannada (India)	Y	General (FTF interview)	N = 381	Random	Interview by a neurologist based on ICHD-2	Within 3–6 weeks
	Herekar et al. 2013	Migraine totally	Urdu (Pakistan)	Y	Healthcare and family (FTF interview)	N = 180 Age: 39.4 ± 14.2 F: 53.9%	Convenience	Clinical diagnosis by a neurologist based on ICHD-2	At the same time
POEM	Kaiser et al. 2019	Migraine totally MO MA	English	Original	Healthcare and community (Self-administered)	N = 90 patients and controls Age: 41–42 ± 6–7 F: 68–87%	Case-control	Clinical diagnosis by a neurologist based on ICHD-2	The gold standard is 5–9 years earlier

**Abbreviations:** CM, chronic migraine; DHD, diagnostic headache diary; EM, episodic migraine; F, female; FMSQ, Finnish migraine-specific questionnaire; FTF, face-to-face; ICHD, international classification of headache disorders; IIBQ, Italian ICHD-II-based questionnaire; MA, migraine with aura; Migraine totally, migraine without aura or with aura, or both; MO, Migraine without aura; MSMDQ, Michel's standardized migraine diagnosis questionnaire; N, no; SAHQ, self-administered headache questionnaire; SHQ, structured headache questionnaire; TTH, tension-type headache; VARS, visual aura rating scale; Y, yes. **Notes:** ^†^ ICHD-II codes.

The HARDSHIP questionnaire has been validated by 4 studies, 33 34 35 36 the ID-migraine 17 18 19 and MS-Q 21 22 23 by 3 each, and the visual aura rating scale has been validated by 2 studies. 38 39 All of the other tools have been validated by a single study.

### Study description

Among included research papers, 10 studies conducted the validation by enrolling a general population sample, 20 26 28 29 30 31 33 35 36 43 2 by enrolling university students, 18 25 14 by enrolling patients, 19 21 22 23 24 27 32 34 37 38 39 40 41 42 and 1 by enrolling workers. 17 Approximately 37% (10 out of 27) of studies involved probability sampling or census, 19 21 23 24 33 35 36 40 42 43 whereas the remainder involved nonprobability sampling. In sum, 17,198 individuals took part in the 27 validation studies, with sample sizes ranging from 49 26 to 9,346. 21 The mean age varied between 22.03 18 and 58.4, 31 notwithstanding 11 studies which failed to provide this information. The percentage of female patients was higher than that of males among studies reporting sex ratios.

The 27 validation studies covered 17 languages, with English being the most frequent one. 26 28 37 41 Among cross-cultural works that required translation, adaptation, and validation, 8 studies implemented a backward-translation verification, 17 23 33 34 35 36 39 43 whereas 4 studies did not. 18 19 25 26 A great number of studies ( *n* = 15) administered migraine diagnostic tools through self-completed questionnaires; 20 21 22 23 25 27 28 29 30 31 32 37 39 40 41 9 of them administered tools through interviews by headache experts or trained interviewers, 17 26 33 34 35 36 38 42 43 and the remaining 3 works did not specify how the validations were conducted. 18 19 24 The reference standard of the included studies was a clinical diagnosis based on the ICHD, editions 1, 2, 3β, or 3, depending on when the validations were completed. The time interval between tool diagnosis and the reference standard was less than 1 month in 11 studies, 17 19 21 22 23 25 32 34 38 39 42 whereas in others it was more than 1 month or was not mentioned.

### Quality assessment

**Figure 2:**
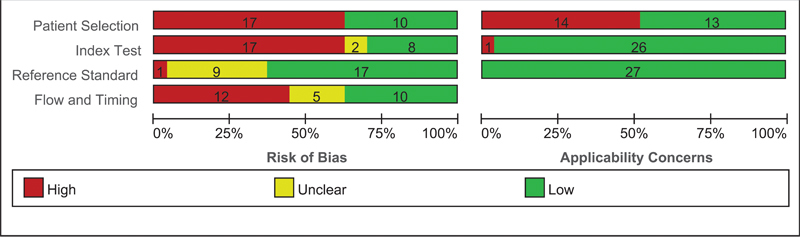
Cumulative bar plot of included studies' risks of bias and applicability concerns.

A summary table of methodological quality assessments for each study is presented in Table 3 . Overall, all studies are “at risk of bias,” with 63% of them related to the participant selection domain, 17 18 20 22 25 26 27 28 29 30 31 32 34 37 38 39 41 70.4% to the index test domain, 18 19 20 21 22 23 24 25 27 28 29 30 31 32 37 38 39 40 41 37% to the reference standard domain, 19 20 22 23 28 31 37 38 40 41 and 63% to the flow and timing domain. 18 20 24 26 27 28 29 30 31 33 35 36 37 40 41 42 43 Moreover, 51.9% of studies were identified as having “concerns regarding applicability” 19 21 22 23 24 27 32 34 37 38 39 40 41 42 , with the domain of participant selection being the most dominant cause. Figure 2 depicts the cumulative bar plot of included studies' risks of bias and applicability concerns.

**Table 3:** Methodological quality assessment for each study according to the QUADAS-2

Tool	Study reference	Risk of bias					Concerns regarding applicability				Quality
		Patient selection	Index test	Reference standard	Flow and timing	Overall	Patient selection	Index test	Reference standard	Overall	
ID-Migraine	Siva et al. 2008	**☹**	**☺**	**☺**	**☺**	At risk	**☺**	**☺**	**☺**	Low concern	MQ
	Wang et al. 2015	**☹**	**☹**	**☺**	**☹**	At risk	**☺**	**☺**	**☺**	Low concern	LQ
	Csépány et al. 2018	**☺**	**?**	**?**	**☺**	At risk	**☹**	**☺**	**☺**	Concerns	LQ
Extended version of ID-migraine	Streel et al. 2015	**☹**	**☹**	**?**	**?**	At risk	**☺**	**☺**	**☺**	Low concern	LQ
MS-Q	Láinez et al. 2005	**☹**	**☹**	**?**	**☺**	At risk	**☹**	**☺**	**☺**	Concerns	LQ
	Láinez et al. 2010	**☺**	**☹**	**☺**	**☺**	At risk	**☹**	**☺**	**☺**	Concerns	LQ
	Delic et al. 2018	**☺**	**☹**	**?**	**☺**	At risk	**☹**	**☺**	**☺**	Concerns	LQ
Simple questionnaire	Gervil et al. 1998	**☺**	**?**	**☺**	**☹**	At risk	**☹**	**☺**	**☺**	Concerns	LQ
MSMDQ	Rueda-Sánchez and Díaz-Martínez, 2004	**☹**	**☹**	**☺**	**☺**	At risk	**☺**	**☺**	**☺**	Low concern	LQ
SHQ	el-Sherbiny et al. 2017	**☺**	**☺**	**☺**	**☹**	At risk	**☺**	**☺**	**☺**	Low concern	MQ
VARS	Eriksen et al. 2005	**☹**	**☹**	**☹**	**☺**	At risk	**☹**	**☺**	**☺**	Concerns	LQ
	Kim et al. 2022	**☹**	**☹**	**☺**	**☺**	At risk	**☹**	**☺**	**☺**	Concerns	LQ
DHD	Phillip et al. 2007	**☹**	**☺**	**☺**	**☹**	At risk	**☺**	**☺**	**☺**	Low concern	LQ
DMQ3	Kirchmann et al. 2006	**☹**	**☹**	**☺**	**☹**	At risk	**☹**	**☹**	**☺**	Concerns	LQ
FMSQ	Kallela et al. 2001	**☺**	**☹**	**?**	**?**	At risk	**☹**	**☺**	**☺**	Concerns	LQ
ID-CM	Lipton et al. 2016	**☹**	**☹**	**?**	**?**	At risk	**☺**	**☺**	**☺**	Low concern	LQ
LUMINA	Van Oosterhout et al. 2011	**☹**	**☹**	**?**	**?**	At risk	**☹**	**☺**	**☺**	Concerns	LQ
HUNT	Hagen et al. 2000	**☹**	**☹**	**☺**	**☹**	At risk	**☺**	**☺**	**☺**	Low concern	LQ
HUNT3	Hagen et al. 2010	**☹**	**☹**	**☺**	**☹**	At risk	**☺**	**☺**	**☺**	Low concern	LQ
HUNT4	Hagen et al. 2019	**☹**	**☹**	**?**	**☹**	At risk	**☺**	**☺**	**☺**	Low concern	LQ
SAHQ	Fritsche et al. 2007	**☹**	**☹**	**☺**	**☺**	At risk	**☹**	**☺**	**☺**	Concerns	LQ
IIBQ	Abrignani et al. 2012	**☺**	**☺**	**☺**	**☹**	At risk	**☹**	**☺**	**☺**	Concerns	LQ
HARDSHIP	Ayzenberg et al. 2011	**☺**	**☺**	**☺**	**☹**	At risk	**☺**	**☺**	**☺**	Low concern	MQ
	Yu et al. 2011	**☺**	**☺**	**☺**	**?**	At risk	**☺**	**☺**	**☺**	Low concern	MQ
	Rao et al. 2012	**☺**	**☺**	**☺**	**☹**	At risk	**☺**	**☺**	**☺**	Low concern	MQ
	Herekar et al. 2013	**☹**	**☺**	**☺**	**☺**	At risk	**☹**	**☺**	**☺**	Concerns	MQ
POEM	Kaiser et al. 2019	**☹**	**☹**	**?**	**☹**	At risk	**☹**	**☺**	**☺**	Concerns	LQ

**Abbreviations:** DHD, diagnostic headache diary; FMSQ, Finnish migraine-specific questionnaire; IIBQ, Italian ICHD-II-based questionnaire; LQ, low quality; MQ, moderate quality; MSMDQ, Michel's standardized migraine diagnosis questionnaire; SAHQ, self-administered headache questionnaire; SHQ, structured headache questionnaire; VARS, visual aura rating scale.

### Diagnostic accuracy

Table 4 gives the diagnostic accuracy of these tools for migraine diagnosis. Due to the fact that the majority of studies were of poor quality, caution should be exercised when considering pooled data; thus, no metanalysis was performed. The sensitivity spanned from 24 29 to 100%, 42 while the specificity spanned from 29 26 to 100%. 20 29 40 42 According to the GBD criteria, diagnostic tools for migraine with both sensitivity and specificity ≥ 70% are desirable. 16 In included studies, 19 studies that validated 14 tools exhibited sensitivity and specificity levels above 70%, 17 20 21 22 23 24 27 28 32 33 34 36 37 38 39 40 41 42 43 with 3 of them reporting both above 90%. 27 38 42 However, due to the different cutoffs for migraine-positive diagnosis among the tools, a direct comparison of diagnostic accuracy was challenging.

**Table 4:** Summary of studies reporting on the diagnostic accuracy of migraine diagnostic tools in non-clinical settings

Tool	Study references	Aim	Sensitivity (%, 95% CI)	Specificity (%, 95% CI)	PPV (%, 95% CI)	NPV (%, 95% CI)
**ID-Migraine**	**Siva et al. 2008**	**Migraine totally**	**70.9 (65.1–75.9)**	**79.1 (72.9–83.7)**	**78.3**	**71.9**
	Wang et al. 2015	Migraine totally	84 (75–90)	64 (59–68)	33	95
	Csépány et al. 2018	Migraine totally	95 (92–97)	42 (31–55)	88 (84–91)	65 (50–78)
Extended version of ID- migraine	Streel et al. 2015	Migraine totally MA	Migraine totally: 87.5 (74.3–100) MA: 83.3 (53.5–100)	Migraine totally: 100 (91.4–100) MA: 96.7 (92.3–100)	Migraine totally: 100 (82.4–100) MA: 71.4 (38–100)	Migraine totally: 93.5 (86.3–100) MA: 98.3 (95.1–100)
MS-Q	Láinez et al. 2005	Migraine totally	93 (87–99)	81 (72–91)	83% (75–91%)	92 (85–99)
	Láinez et al. 2010	Migraine totally	82 (81–84)	98 (98–99)	95% (94–96%)	94 (93–95)
	Delic et al. 2018	Migraine totally	80 (74.7–84.6)	87.2 (81.1–91.9)	91% (87.1–93.8%)	72.9 (67.8–77.6)
Simple questionnaire	Gervil et al. 1998	Migraine totally	85	81	49%	86
MSMDQ	Rueda-Sánchez and Díaz-Martínez, 2004	Migraine totally	37.9 (25.8, 51.7)	99.1 (94.4–100)	95.7 (76–99.8)	75.5 (67.6–82.1)
SHQ	el-Sherbiny, 2017	EM CM	EM: 86 (78–97) CM: 71 (52–99)	EM: 94 (86–98) CM: 98 (82–100)	EM: 86 (69–98) CM: 78 (63–96)	EM: 92 (87–99) CM: 96 (82–100)
VARS	Eriksen et al. 2005	MA	91 (86–95)	96 (91–100)	99	78
	Kim et al. 2022	MA	96.4 (84.5–99.6)	79.5 (72.9–85)	58.2 (51.1 - 65)	98.7 (95–99.7)
DHD	Phillip et al. 2007	Migraine totally	97%	29	85	75
DMQ3	Kirchmann et al. 2006	Migraine totally MO MA MO + MA	Migraine totally: 99 (97–100) MO: 91 (81–100) MA: 77 (63–90) MO + MA: 63 (48–78)	Migraine totally: 86 (75–97) MO: 93 (88–98) MA: 88 (82–94) MO + MA: 92 (87–97)	Migraine totally: 96 MO: 79 MA: 70 MO + MA: 73	Migraine totally: 97 MO: 97 MA: 91 MO + MA: 88
FMSQ	Kallela et al. 2001	MO MA MO + MA	MO: 61 MA: 89 MO + MA: 74	MO: 100 MA: 96 MO + MA: 97	MO: 100 MA: 85 MO + MA: 91	MO: 89 MA: 97 MO + MA: 90
ID-CM	Lipton et al. 2016	Migraine totally CM	Migraine totally: 83.5 CM: 80.6	Migraine totally: 88.5 CM: 88.6	Migraine totally: 62.2 CM: 75	Migraine totally: 96 CM: 91.5
LUMINA	Van Oosterhout et al. 2011	MA	86	75	74	86
HUNT	Hagen et al. 2000	Migraine totally	24	100	100	62
HUNT3	Hagen et al. 2010	Migraine totally MA	Migraine totally: 51 (45–97) MA: 50 (44–56)	Migraine totally: 95 (92–98) MA: 95 (93–97)	Migraine totally: 61 MA: 43	Migraine totally: 93 MA: 98
HUNT4	Hagen et al. 2019	Migraine totally MA	Migraine totally: 48 (32–63) MA: 33 (11–55)	Migraine totally: 93 (90–96) MA: 94 (91–97)	Migraine totally: 63 MA: 37	Migraine totally: 89 MA: 93
SAHQ	Fritsche et al. 2007	Migraine totally Migraine + TTH	Migraine totally: 73.2 (63.2–81.7) Migraine + TTH: 62.1 (42.3–79.3)	Migraine totally: 96.1 (92.2–98.4) Migraine + TTH: 97.8 (94.9–99.3)	Migraine totally: 91 (82.4–96.3) Migraine + TTH: 78.3 (56.3–92.5)	Migraine totally: 87 (81.5–91.3) Migraine + TTH: 95.3 (91.8–97.6)
IIBQ	Abrignani et al. 2012	MO MA (1.2.1, 1.2.2)† MA (1.2.3)†	MO: 100 MA (1.2.1, 1.2.2): 100 MA (1.2.3): 50 (36–64)	MO: 93.3 (86–100) MA (1.2.1, 1.2.2): 100 MA (1.2.3): 100	MO: 97 MA (1.2.1, 1.2.2): 100 MA (1.2.3): 100	MO: 100 MA (1.2.1, 1.2.2): 100 MA (1.2.3): 98
HARDSHIP	Ayzenberg et al. 2011	Migraine totally	76.9 (68.1–84)	82.4 (77.8–86.1)	69.4 (61.5–75.8)	87.3 (82.4–91.2)
	Yu et al. 2011	Migraine totally	83.3 (79.7–87)	99 (97.5–99.8)	83% (70–96.7)	99 (97.5–99.8)
	Rao et al. 2012	Migraine totally	63 (52–72)	85 (81–89)	55 (45–65)	89 (85–92)
	Herekar et al. 2013	Migraine totally	74	87	60	92
POEM	Kaiser et al. 2019	Migraine totally MO MA	Migraine totally: 83 MO: 59 MA: 70	Migraine totally: 90 MO: 84 MA: 93	Migraine totally: 94 MO: 63 MA: 84	Migraine totally: 74 MO: 81 MA: 86

**Abbreviations:** CM, chronic migraine; DHD, diagnostic headache diary; EM, episodic migraine; FMSQ, Finnish migraine-specific questionnaire; IIBQ, Italian ICHD-II-based questionnaire; MA, migraine with aura; Migraine totally, migraine without aura or with aura, or both; MO, migraine without aura; MSMDQ, Michel's standardized migraine diagnosis questionnaire; SAHQ, self-administered headache questionnaire; SHQ, structured headache questionnaire; TTH, tension-type headache; VARS, visual aura rating scale. **Notes:** ^†^ ICHD-II codes.

Whether a study has good methodological quality determines if it can generate unbiased estimates of diagnostic accuracy. 14 It should be noted that no tool has been supported by high-quality evidence, regarding the use in nonclinical circumstances. The diagnostic accuracy of the ID-migraine, 17 structured headache questionnaire, 43 and HARDSHIP questionnaire 33 34 35 36 have been supported by moderate-quality evidence, with satisfactory sensitivity and specificity. Of them, the HARDSHIP questionnaire was the most extensively validated. The evidence of the remaining 16 tools for use in the nonclinical population has been of poor quality; thus, their diagnostic performance should be generalized with caution.

## DISCUSSION

### Summary of findings

This systematic review identified 27 studies that validated 19 tools currently used for migraine diagnosis without the need for doctor consultation. For use in nonclinical settings, no tool has been supported by high-quality evidence; the diagnostic accuracy of 3 tools (the HARDSHIP questionnaire, ID-migraine, and structured headache questionnaire) has been supported by moderate-quality evidence, and the remaining tools only have studies which provided poor-quality evidence. The quality assessment findings are largely consistent with a previously published systematic review focusing on chronic headache disorders. 7

The ID-migraine, structured headache questionnaire, and HARDSHIP questionnaire have demonstrated satisfactory diagnostic accuracy in nonclinical settings, supported by moderate-quality evidence. The HARDSHIP questionnaire has been the most widely validated of all. In poor-quality studies, evidence for diagnostic accuracy is limited by certain shortcomings; as these quality issues have the potential to impair the robustness of these studies, we caution against extrapolating outcomes. Considering the evidence mentioned above, it is suggested that the HARDSHIP questionnaire is the optimal choice for diagnosing migraine in nonclinical settings to date.

### Public health significance

Underdiagnosis and undertreatment of migraines are common, especially due to its trivialization. 44 Furthermore, migraine is stigmatized, and people typically conceal migraine attacks due to guilt about missing work and fear of workplace retaliation and dismissal. 45 Patients themselves are also an obstacle to better care, usually due to mistrust in doctors' abilities. However, this could be related to the fact that few individuals contact their physicians regarding this matter, and, hence, are unable to benefit from medical expertise or available treatments. 46

This necessitates the advancement of a migraine diagnostic tool, allowing for effective case detection in nonclinical contexts, such as the community or workplace. The reason for guidelines to suggest that migraine diagnostic tools designed for use in nonclinical settings should be validated in populations in these settings against the “gold standard” 11 is because the diagnostic accuracy of a tool may vary with the population being tested, target contexts, and many other factors. 14 The present systematic review provides sufficient details about existing migraine diagnostic tools for application in nonclinical settings. The public health significance of this review is important, since we anticipate that it can inform decisions on how to choose and utilize these tools for researchers and practitioners, to promote earlier diagnosis, initiation of appropriate treatment, and reduction in disease burden.

### Quality issues of existing evidence and recommendations for future research

High risks of bias and/or applicability concerns in methodological quality are important limitations of the robustness of a study. 14 Among included studies, self-administration of a diagnostic tool, delay between diagnostic tool and reference standard, lack of representativeness of nonclinical populations, absence of blindness, and poor study flow are the leading sources of risk of bias, while participant selection is the leading source of concerns regarding applicability in nonclinical settings.

Self-administered diagnosis, which was frequently employed in the included studies, 20 21 22 23 25 27 28 29 30 31 32 37 39 40 41 may introduce information bias because it does not help with question clarification, low literacy assistance, or participant engagement when compared with face-to-face or telephone interviews. 11 Furthermore, as migraine progression between tool diagnosis and “gold standard” is likely to vary, the time interval between them should preferably be less than 1 month. 11 In terms of sampling methods, some studies enrolled patients from clinical settings, 19 21 22 23 24 27 32 34 37 38 39 40 41 42 preliminarily screen-positive subjects, 17 41 or case-control designs, 32 37 who had more typical or more extreme symptoms, resulting in inflated sensitivity and specificity estimations. 14 A further quality issue is that several studies 17 18 20 25 26 28 29 30 31 recruited an unrepresentative convenience/volunteer sample, despite being from nonclinical circumstances, which could introduce selection bias. Also, a low participation rate (< 70%) cannot guarantee representativeness. 11 Next, in studies where no blindness existed between the tool's diagnosis and the reference standard, 19 20 22 23 28 31 37 38 40 41 the interpretation of tools' results could be influenced by knowledge of the reference standard results. 15 Additionally, not all participants received the same reference standard in several studies: some had a face-to-face clinical interviews with a neurologist, while others had a telephone interview, 18 26 42 which may lead to biased diagnostic performance. 47

Even though all of the included tools were reported to be applicable to nonclinical settings, we discovered a primary applicability concern with respect to participant selection: participants in 14 validation studies 19 21 22 23 24 27 32 34 37 38 39 40 41 42 were healthcare users, who were more likely to be disabled and had rehearsed their medical histories. They did not match our target nonclinical population, resulting in a lack of external validity. 11 Furthermore, cross-cultural validation was lacking for some tools. 18 19 25 26

This systematic review seeks to provide relevant and up-to-date information on the use of migraine diagnostic tools in nonclinical contexts, as well as to uncover knowledge gaps. A crucial next step is more high-quality validation studies in diverse samples in the nonclinical population against the “gold standard.”

It is suggested that future studies enhance their methodological quality, with particular attention to interview administration, time interval, sampling methods, response rate, blindness, and study flow. The most important is that tools should be validated among the general population. Moreover, the diversity of the global population, particularly in terms of ethnicity, culture, and language, warrants cross-cultural validation.

### Strengths and limitations

This is, to our best knowledge, the first systematic review of studies validating migraine diagnostic tools applicable to nonclinical settings. Multidisciplinary workgroup collaboration, a combination of comprehensive search strategies for multiple electronic databases and manual searches, an explicit and systematic methodology, and rigorous quality assessment are the strengths of our systematic review.

However, this work has several limitations. The first is the inclusion of only English-language peer-review articles. Certain studies were also excluded because they did not report diagnostic accuracy; however, if these authors provided specific data, such as prevalence, we were able to calculate some outcomes. Also, the probability of publication bias cannot be ruled out. Following that, quantitative synthesis and data comparison were not easy due to the quality of evidence and heterogeneity of the included studies. The various cut-off levels, which were a compromise between false positives and false negatives, resulted in non-comparability among studies.

In conclusion, up to now, the HARDSHIP questionnaire is the optimal choice for diagnosing migraine in nonclinical settings, with satisfactory diagnostic accuracy supported by moderate methodological quality. The significance of this study is to inform tool selection decisions for researchers and practitioners, contributing to earlier diagnosis, treatment initiation, and disease burden reduction. For better migraine case identification in nonclinical settings, future high-quality validation studies among varied nonclinical population groups are encouraged, with a methodological emphasis on interview administration, time interval, sampling methods, response rate, blindness, and study flow.
