# Supplementary material for: Validation studies on migraine diagnostic tools for use in nonclinical settings: a systematic review
Source: Arq Neuropsiquiatr. 2022 Oct 27;81(4):399–412. doi: 10.1055/s-0042-1756490 (PMC10169234; doi:10.1055/s-0042-1756490)
Supplement: Supplementary file 1 — Supplementary Material [file 10-1055-s-0042-1756490-s220093.pdf]

Supplementary Material

Supplementary Table S1 Search strategies

No	Search string	Result
**PubMed**		
#1	(migraine*) OR (migraine disorder [MeSH Terms])	43,443
#2	((“diagnostic techniques and procedures” [MeSH Terms]) OR (“diagnosis” [MeSH Terms])) OR (diagnos* OR screen* OR prevalence OR exam* OR interview*)	15,013,666
#3	(((tool* OR questionnaire* OR measure* OR scale* OR instrument* OR survey* OR algorithm* OR app application* OR model* OR system*)) OR (surveys and questionnaires [MeSH Terms])) OR (“algorithms” [MeSH Terms])	13,037,532
#4	(“predictive value of tests” [MeSH Terms]) OR (Sensitivity and Specificity [MeSH Terms]) OR (sensitiv* OR specific* OR valid* OR accura* OR (predict* AND value*))	6,452,155
#5	((((((“adolescent” [MeSH Terms]) OR (“child” [MeSH Terms])) OR (“pediatrics” [MeSH Terms])) OR (adolescent* [Title/Abstract])) OR (child* [Title/Abstract])) OR (pediatric* [Title/Abstract])) OR (pediatric* [Title/Abstract])	3,801,688
#6	(#1 AND #2 AND #3 AND #4) NOT #5	2,827
#7	Limited to English	2,697
**Medline**		
1	(MH “Migraine Disorders + ”) OR (TI migraine#) OR (AB migraine#)	42,794
2	(MH “Diagnostic Techniques and Procedures + ”) OR (MH “Diagnosis + ”) OR (TI (diagnos* OR screen* OR prevalence OR exam* OR interview*)) OR (AB (diagnos* OR screen* OR prevalence OR exam* OR interview*))	13,249,451
3	(MH “Surveys and Questionnaires + ”) OR (TI (tool* OR questionnaire* OR measure* OR scale* OR instrument* OR survey*)) OR (AB (tool* OR questionnaire* OR measure* OR scale* OR instrument* OR survey*))	9,753,893
4	(MH “Predictive Value of Tests + ”) OR (MH “Sensitivity and Specificity + ”) OR (TI (sensitiv* OR specific* OR valid* OR accura* OR (predict* n2 value*))) OR (AB (sensitiv* OR specific* OR valid* OR accura* OR (predict* n2 value*)))	5,886,848
5	(MH “Pediatrics + ”) OR (TI (pediatric* OR pediatric*)) OR (AB (pediatric* OR pediatric*)) OR (MH “Child + ”) OR (TI child*) OR (AB child*) OR (MH “Adolescent”) OR (TI Adolescent#) OR (AB Adolescent#)	3,796,960
6	(1 AND 2 AND 3 AND 4) NOT 5	2,022
7	Limited to English	1,932
**Web of Science**		
#1	TS = (migraine$)	52,068
#2	TS = (diagnos* OR screen* OR prevalence OR exam* OR algorithm* OR app application* OR model* OR system*)	9,995,105
#3	TS = (tool* OR questionnaire* OR measure* OR scale* OR instrument* OR survey* OR interview*)	26,232,161
#4	TS = (sensitiv* OR specific* OR valid* OR accura* OR (predict* NEAR/2 value*))	10,411,863
#5	TS = (Adolescent$ OR child* OR pediatric*)	2,426,420
#6	(#1 AND #2 AND #3 AND #4) NOT #5	2,687
#7	Limited to English	2,584

 © 2022. Academia Brasileira de Neurologia. All rights reserved.

Supplementary Table S2 Quality thresholds of population-based studies on headaches according to the GBD study

Domain	Less desirable	Threshold
Recall period	Other than 1-year prevalence	1-year prevalence
Representativeness of sample	Selected population	General population or community-based sample
Sampling method	Not stated; Other sampling methods	Random sampling
Response rate	Not stated; $< 70\%$	$\geq 70\%$
Interviewer	Not stated; self-administered; telephone or FTF interview by untrained or unspecified interviewer(s)	FTF interview with headache expert or trained interviewer
Validation	Instruments not validated; sensitivity and specificity $< 70\%$; validated only in screen-positive subsample, or in clinic or unspecified sample, but sensitivity and specificity $\geq 70\%$	Validated in target population or similar and sensitivity and specificity $\geq 70\%$
Compared diagnostic criteria	Not stated; Other than ICHD (or reasonable modification)	ICHD (or reasonable modification)

Abbreviation: FTF, face-to-face; GBD, global burden of disease; ICHD, international classification of headache disorders.
Notes: Adapted from GBD Neurology Collaborators (2019).

 © 2022. Academia Brasileira de Neurologia. All rights reserved.

Supplementary Table S3 Review-specific signaling questions for quality assessment

Domain	Rating	Signaling question
**Risk of bias**		
Participant selection	☺	Random sample AND response 70–100%
	☹	No (or failed) attempt to secure representativeness OR Response < 70%
	?	Not stated
Index test	☺	FTF interview with headache expert or trained interviewer AND Index test results interpreted without knowledge of the results of the reference standard
	☹	Self-administered (unsupervised) questionnaire OR Telephone or FTF interview by untrained or unspecified interviewer(s) OR Index test results interpreted with knowledge of the results of the reference standard
	?	Not stated
Reference standard	☺	All diagnoses made in FTF or telephone interviews by headache expert(s) based on ICHD AND The reference standard results interpreted without knowledge of the results of the index test
	☹	Diagnoses not made in FTF or telephone interviews by headache expert based on ICHD OR The reference standard results interpreted with knowledge of the results of the index test
	?	Not stated
Flow and timing	☺	A maximum of 1 month interval between index test(s) and reference standard AND All patients receive a same reference standard AND All patients included in the analysis
	☹	Above 1 month interval between index test(s) and reference standard OR Not all patients receive a same reference standard (among subsample) OR Not all patients included in the analysis
	?	Not stated
Overall	If a study is judged as "low" on all domains relating to bias, then it is appropriate to have an overall judgment of "low risk of bias" for that study. If a study is judged "high" or "unclear" in 1 or more domains, then it may be judged "at risk of bias."	
**Concerns regarding applicability**		
Participant selection	☺	General population, or defined population in non-clinical settings
	☹	Users of healthcare resources
Index test	☺	The index test, its conduct, or interpretation accord with the review question
	☹	The index test, its conduct, or interpretation differ from the review question
Reference standard	☺	All diagnoses made in FTF or telephone interviews by headache expert(s) based on ICHD
	☹	Diagnoses not made by headache expert(s) based on ICHD
Overall	If a study is judged as "low" on all domains relating to applicability, then it is judged as "low application concern." If a study is judged "high" or "unclear" in 1 or more domains, then it is appropriate to have a judgement of "application concerns."	
**Quality**		
High quality		Overall "low risk of bias" in combination with overall "low concern regarding applicability."
Moderate quality		One domain receiving "high" or "unclear" risk of bias in combination with overall "low concern regarding applicability."
Poor quality		All other rating combinations.

Abbreviation: FTF, face-to-face; ICHD, international classification of headache disorders.

 © 2022. Academia Brasileira de Neurologia. All rights reserved.
